# The Prevalence and Antimicrobial Susceptibility of Methicillin-Resistant Staphylococcus aureus Before and After the COVID-19 Pandemic in a Tertiary Saudi Hospital

**DOI:** 10.7759/cureus.54809

**Published:** 2024-02-24

**Authors:** Omar B Ahmed, Fayez S Bahwerth, Radi Alsafi, Eman A Elsebaei, Gamal T Ebid, Abdulrhaman Theyab, Hamza Assaggaf

**Affiliations:** 1 Environmental and Health Research, Umm Al-Qura University, Makkah, SAU; 2 Laboratory, King Faisal Hospital, Makkah, SAU; 3 Laboratory Medicine, Faculty of Applied Medical Sciences, Umm Al-Qura University, Makkah, SAU; 4 Medical Microbiology, Egypt Healthcare Authority, Cairo, EGY; 5 Laboratory Medicine, Security Forces Hospital, Makkah, Makkah, SAU; 6 National Cancer Institute, Cairo University, Cairo, EGY; 7 Department of Laboratory and Blood Bank, Security Forces Hospital, Mecca, Makkah, SAU; 8 Collage of Medicine, Al-Faisal University, Riyadh, SAU; 9 Laboratory Medicine/Public Health, Umm Al-Qura University, Makkah, SAU

**Keywords:** covid-19, makkah, mssa, mrsa, s. aureus

## Abstract

Background: Methicillin-resistant *Staphylococcus aureus* (MRSA) has become a major public health problem all over the world. After the 2019 coronavirus illness (COVID-19), the pandemic may have influenced research priorities and resource allocation, potentially affecting the ability to monitor MRSA trends.

Aims: The study aimed to evaluate the prevalence of *S. aureus,* including MRSA infections, and their antimicrobial susceptibilities over the years 2019 and 2020 in a tertiary hospital in Makkah City, KSA.

Methodology: A total of 2128 and 1515 laboratory (lab) samples were collected during the years 2019 and 2020, respectively. From these samples, the prevalence of *S. aureus,* including MRSA, and their antibiotic susceptibility were identified using standard, automated, and molecular microbiological methods.

Results: The present study shows that the lab prevalence of all S.* aureus* during 2019 was found to be 35.5%, of which MRSA was 44.8%. During 2020, the frequency of *S. aureus* strains was 16%, of which MRSA was 41.2%. The most common MRSA isolated during both years were colonizing pus swabs and urine samples. The results showed that MRSA susceptibility against antimicrobial agents in 2019 was as follows: vancomycin (100%), linezolid (100%), trimethoprim-sulfamethoxazole (88%), and doxycycline (34.2%). The MRSA strains isolated during 2020 were as follows: vancomycin (100%), linezolid (96%), trimethoprim-sulfamethoxazole (100%), and doxycycline (24.3%). There was no significant difference in the incidence and antimicrobial resistance rates of MRSA over the two years.

Conclusion: It was concluded that the prevalence rates of MRSA have not increased in 2020 when compared to 2019. Vancomycin, linezolid, trimethoprim-sulfamethoxazole, and doxycycline remain susceptible to the positive collected MRSA strains. There was no significant difference between the prevalence and antimicrobial resistance rates of MRSA between 2019 and 2020. Continued research efforts are needed to address this persistent public health threat. Strategies to control the spread of MRSA should include early detection of MRSA and surveillance, even during pandemics.

## Introduction

Methicillin-resistant *Staphylococcus aureus* (MRSA) infection has become a major public health problem all over the world. It is correlated with increased morbidity and mortality compared to other pathogenic bacteria. MRSA is a type of bacteria that is resistant to many antibiotics. The prevalence of MRSA in Saudi Arabia is reported as 25-55%, with the dominance of community-acquired MRSA (CA-MRSA) (23-30%), while hospital-acquired MRSA (HA-MRSA) represents 54% of all nosocomial infections caused by *S. aureus* clinical isolates. COVID-19 has been a pandemic since it was first discovered in December 2019. The emergence of the COVID-19 pandemic has not directly altered the characteristics of MRSA itself, such as cell-wall-associated virulence determinants and a broad spectrum of extracellular proteins, but it may have influenced certain factors, such as bacterial and fungal infection in COVID-19 patients, inappropriate prescribing and use of antibiotics, increased use of biocides, and the impact of compromised healthcare services in the rise of COVID-19 [1]. In Ghana, MRSA carriage rates range from 25% to 50%; people who inject drugs (PWID), have insulin-dependent diabetes, have dermatological diseases (Impetigo), have indwelling intravascular catheters, or work in healthcare have greater rates of incidence than the general population [2]. Before COVID-19, challenges in healthcare systems were limited to the treatment of antibiotic-resistant infections like MRSA. After COVID-19, the strain on healthcare systems during the pandemic might have influenced the management of MRSA cases, potentially diverting resources and attention [3]. Over the past decade (before COVID-19), the percentage of *S. aureus* clinical specimens that are methicillin-resistant has been steadily increasing in many Nordic countries, with 61.8 new cases per 100,000 individuals in 2016 as the highest, where rates were highest in inpatient lower respiratory specimens and lowest in outpatient skin and soft tissue specimens [4]. After COVID-19, the pandemic may have influenced research priorities and resource allocation, potentially affecting the ability to monitor MRSA trends. Epidemiological studies indicate that bacterial co-infections are the primary contributor to the higher mortality of COVID-19 rather than the virulence of the virus itself. S. aureus is a common bacterial infection that causes pneumonia [5]. It has been reported that secondary pneumonia may be brought on by several bacteria, including *S. aureus* [6]. Influenza A virus may also be the primary culprit in severe respiratory conditions that cause high rates of morbidity and death. MRSA strains are among the pathogens that have become increasingly common over the past few decades, which has led to issues that have increased the annual prevalence of pneumonia and death (65,000 deaths) in the United States [7,8]. The history of MRSA has been characterized by a series of waves of change since the 1940s [9]. It is appropriate to analyse the advent of MRSA and point out significant turning points for increasing the burden of COVID-19 infection. In Makkah city (the pilgrimage city) in Saudi Arabia, the rate of MRSA was reported at more than 22% [10,11]. The close proximity of large numbers of people in confined spaces facilitates the transmission of various pathogens, like MRSA [10]. In Saudi Arabia, the incidence of MRSA infections has been reported by authors [10,11]; however, few published papers compared the prevalence of *S. aureus* and MRSA strains and their antimicrobial susceptibilities before and after COVID-19 in Saudi Arabia. The study aimed to determine the prevalence of *S. aureus*, including MRSA strains, and their antimicrobial susceptibilities over the years 2019 and 2020 in a tertiary hospital in Makkah city, Saudi Arabia.

## Materials and methods

Study design

A cross-sectional descriptive laboratory-based study was undertaken to determine the prevalence of MRSA in the Security Forces Hospital Makkah (SFHM) and their susceptibilities to antibiotics before and during the COVID-19 pandemic. *S. aureus* was isolated from regular (non-COVID-19) patients who attended SFHM, Makkah, Saudi Arabia, during the years 2019 and 2020. The Institutional Review Board (IRB) in SFHM approved the study with Ref. No. 0329-021219.

Sample collection

Researchers conducted a cross-sectional descriptive laboratory-based study to determine the prevalence of MRSA in the SFHM and its susceptibilities to antibiotics before and during the COVID-19 pandemic. *S. aureus* was isolated from regular (non-COVID-19) patients who attended SFHM, Makkah, Saudi Arabia, during the years 2019 and 2020. The IRB in SFHM approved the study with Ref. no. 0329-021219.

Strains identification

The identities of the strains were confirmed and identified as MRSA using standard microbiological methods such as catalase, the coagulase test (staphylococcus latex test), the cefoxitin disc (FOX) screen test, and the automated VITEK 2 compact marker of mecA/mecC-mediated to define the MRSA strain. According to Ahmed [12], PCR testing was conducted. Briefly, DNA was extracted by the microwave lysis method and then added to detect the resistance mecA gene using specific primers MECA P4 (5' TCCAGATTACAACTTCACCAGG -3') and MECA P7 (5'- CCACTTCATATCTTGTAACG -3'), which were then amplified at a 162-bp fragment. These strains were characterized using antimicrobial susceptibility testing by a commercial microdilution system (VITEK 2 Compact). Antimicrobial susceptibility for antibiotics including moxifloxacin, erythromycin, clindamycin, vancomycin, linezolid, trimethoprim-sulfamethoxazole, gentamicin, nitrofurantoin, and oxacillin was assessed using a specific card for Gram-positive bacteria analyzed with the automated instrument Vitek 2 (Biomerieux SA, Marcy-l'Étoile, France).

Statistical analysis

Statistical analysis was performed using SPSS “Statistical Package for the Social Sciences" (IBM SPSS Statistics V22.0, Armonk, NY). The chi-square test was used to evaluate the relationship between the rates of MRSA infections during the two years. The p-value < 0.05 was considered to be statistically significant.

Ethical approval

The Institutional Review Board in SFHM approved the study (Ref. No. 0329-021219). Informed consent was not required.

## Results

**Figure 1:**
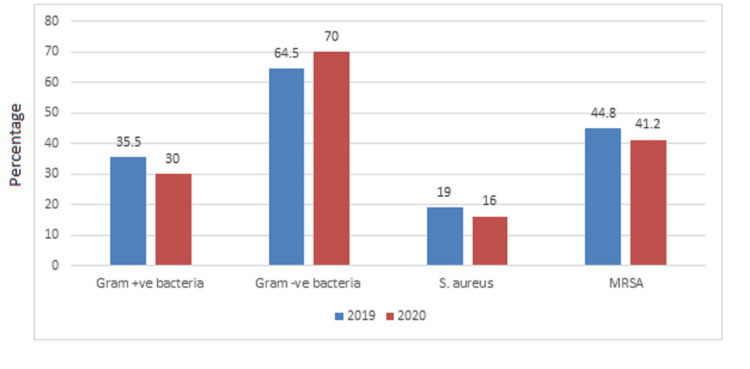
Bacterial incidence rates through the years 2019 and 2020.

Table 1 and Figure 1 show that the frequency of Gram-positive bacteria in 2019 was 756 (35.5%), while Gram-negative bacteria was 1372 (64.5%). The frequency of *S. aureus* in 2019 was 261 (19%), of which MRSA was 44.8% and MSSA was 55.2%. The frequency of Gram-positive bacteria during 2020 was 455 (30%), while Gram-negative bacteria was 1060 (70%). The frequency of *S. aureus* during 2020 was 170 (16%), of which MRSA was 41.2% and MSSA was 58.8%.

**Table 1:** Incidence of Gram-positive bacteria and MRSA through 2019 and 2020. MRSA: methicillin-resistant *S. aureus*; MSSA: methicillin-sensitive *S. aureus.*

Variables	2019	2020
Gram-negative bacteria	1372 (64.5%)	1060 (70%)
Gram-positive bacteria	756 (35.5%)	455 (30%)
Total	2128 (100%)	1515 (100%)
S. aureus	261 (19%)	170 (16%)
MRSA	117 (44.8%)	70 (41.2%)
MSSA	144 (55.2%)	100 (58.8%)

**Figure 2:**
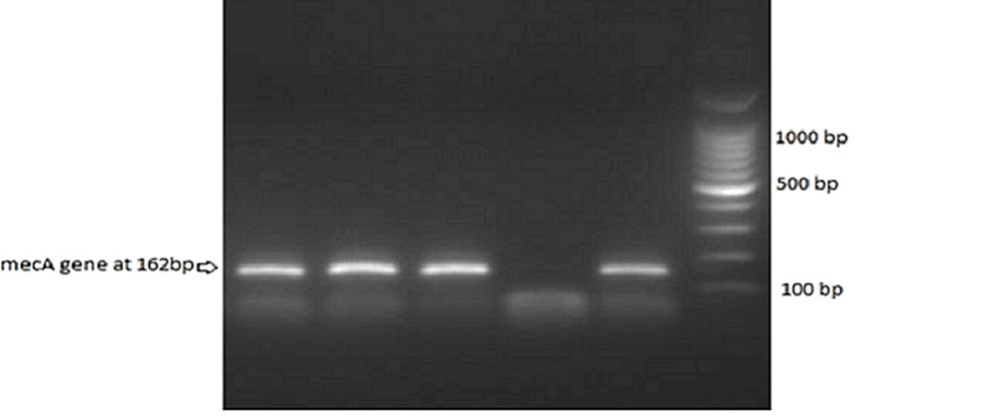
Detection of mecA gene (162 bp) by PCR in MRSA strains. MRSA: methicillin-resistant *S. aureus*; PCR: polymerase chain reaction.

About 33.3% of the *S. aureus* strains isolated during the year 2019 were from pus swabs, followed by blood samples (24.5%) and urine samples (17.24%), while during the year 2020, the majority of the *S. aureus* strains were isolated from pus swabs (47%), followed by blood samples (23%), and UTI samples (12%), as shown in Table 2.

**Table 2:** Distribution of S. aureus according to sample type through the years 2019 and 2020. LRTI: low respiratory tract infection.

Samples	N(%)	
	2019	2020
Blood	64 (24.5%)	39 (23%)
Urine	45 (17.24%)	21 (12%)
LRTI	14 (5.36%)	6 (3.5%)
Pus swabs	87 (33.3%)	80 (47%)
Tissue biopsies	25 (9.6%)	7 (4%)
Body fluids	26 (10%)	17 (10%)
Total	261 (100%)	170 (100%)

**Figure 3:**
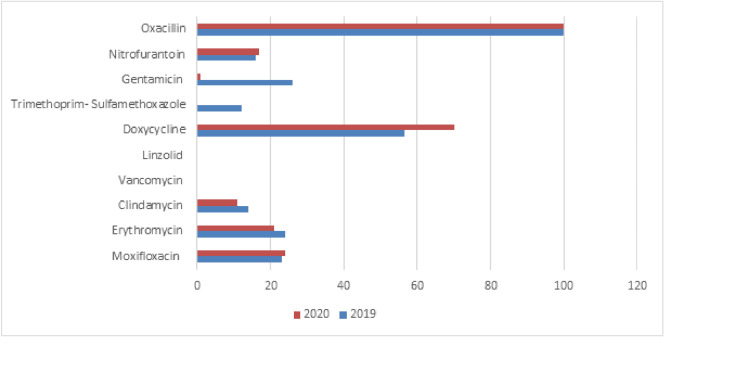
Resistance rates of methicillin-resistant S. aureus isolates.

Table 3 and Figure 3 show the results of the antimicrobial susceptibility assay against MRSA strains. All MRSA isolates showed 100% resistance rates to oxacillin (100%). The results showed that MRSA susceptibility against antimicrobial agents in 2019 was as follows: vancomycin (100%), linezolid (100%), trimethoprim-sulfamethoxazole (88%), and doxycycline (34.2%). The MRSA strains isolated during 2020 were as follows: vancomycin (100%), linezolid (96%), trimethoprim-sulfamethoxazole (100%), and doxycycline (24.3%). There was no significant difference in the incidence and antimicrobial resistance rates of MRSA between the two years (2019 and 2020) (p-value > 0.05).

**Table 3:** Antibiotic susceptibility of methicillin-resistant S. aureus.

Antibiotic	2019			2020		
	Sensitive	Intermediate	Resistant	Sensitive	Intermediate	Resistant
Moxifloxacin	87 (72%)	6 (5%)	27 (23%)	44 (63 %)	9 (13%)	17 (24%)
Erythromycin	82 (68%)	10 (8%)	28 (24%)	49 (70 %)	6 (9%)	15 (21 %)
Clindamycin	96 (80 %)	7 (6%)	17 (14%)	56 (80%)	6 (9%)	8 (11%)
Vancomycin	120 (100%)	0 (0%)	0 (0%)	70 (100%)	0 (0%)	0 (0%)
Linezolid	120 (100%)	0 (0%)	0 (0%)	67 (96%)	3 (4%)	0 (0%)
Doxycycline	41 (34.2%)	11 (9.2%)	68 (56.6%)	17 (24.3%)	4(5.7%)	49 (70%)
Trimethoprim-sulfamethoxazole	106 (88%)	0 (0%)	14 (12%)	70 (100%)	0 (0%)	0 (0%)
Gentamicin	82 (68%)	7 (6%)	31 (26%)	65 (93%)	4 (6%)	1 (1%)
Nitrofurantoin (only in urine) 26 in urine	16 (84%)	0 (0%)	3 (16%)	5 (83%)	0 (0%)	1 (17%)
Oxacillin	0 (0%)	0 (0%)	120 (100%)	0 (0%)	0 (0%)	70 (100%)

## Discussion

The study aimed to evaluate the prevalence of *S. aureus*, including MRSA strains, and their antimicrobial susceptibilities over the years 2019 and 2020 in the SFHM. The results of the present study showed that the frequency of *S. aureus* during 2019 was 756 (35.5%), of which MRSA was 44.8%, and during 2020, the frequency of *S. aureus* was 170 (16%), of which MRSA was 41.2%. The epidemiology of infections caused by MRSA is rapidly changing [13]. In Saudi Arabia, many studies have reported an increase in the incidence of MRSA in recent years. One study [14] found that 55.3% of clinical *S. aureus* isolates were MRSA, whereas earlier studies conducted in the Jeddah hospitals showed a lower prevalence, with only minor variation between 6.5% and 8.9% [15,16]. The present study showed that 33.3% of the *S. aureus* strains isolated during the year 2019 were from pus swabs, followed by blood samples (24.5%) and UTIs (17.24%), while during the year 2020, most of the *S. aureus* strains were isolated from pus swabs (47%), followed by blood samples (23%), and urine samples (12%). Before the COVID-2019 pandemic, many reports in Saudi Arabia reported a variable number of MRSA strains from various body sites in Makkah, Madinah, and Riyadh cities [14,17,18,19]. The results of our study showed that MRSA susceptibility against antimicrobial agents in 2019 was as follows: vancomycin (100%), linezolid (100%), trimethoprim-sulfamethoxazole (88%), and doxycycline (34.2%). The MRSA strains isolated during 2020 were as follows: vancomycin (100%), linezolid (96%), trimethoprim-sulfamethoxazole (100%), and doxycycline (24.3%). All MRSA isolates showed 100% resistance rates to oxacillin (100%), followed by resistance to gentamicin (29%) and erythromycin (24%) during 2019, while during 2020 they expressed resistance to moxifloxacin (98%), erythromycin (21%), nitrofurantoin (17%), and clindamycin (11%). A similar study reported the presence of S*. aureus* bacteremia, of which 29.1% were MRSA, which was almost susceptible to vancomycin, linezolid, and tigecycline [20]. Gram-positive cocci are rarely resistant to linezolid, as isolates with an MIC ≤ 4.0 mg/L are considered susceptible to linezolid, and isolates with an MIC ≥ 8.0 mg/L are resistant. A recent study examined the linezolid susceptibility of 1930 MRSA isolates collected from different regions of the United States; 99.9% were susceptible to linezolid [21]. While vancomycin-resistant *S. aureus* was 0% before 2019 and 0% after 2019, previously, many studies from KSA reported no or very low vancomycin resistance rates among MRSA and *S. aureus* strains. On the other hand, one study showed that MRSA strains were 100% resistant to linezolid [22]. The glycopeptides vancomycin, teicoplanin, and oxazolidinone linezolid have been considered the drugs of choice for the treatment of MRSA infections [23,24]. *S. aureus* represents a part of the human flora and is a common cause of infections (community and nosocomial). It has been reported that the Nordic countries, such as Denmark, Finland, Iceland, Norway, and Sweden, have had a very low prevalence of MRSA, with less than 3% of *S. aureus* bacteremia isolates being MRSA [4]. During influenza seasons, it is possible that *S. aureus* is one of the most commonly causative agents of secondary bacterial infection. In 2020, the viral pandemic caused by severe acute respiratory syndrome coronavirus 2 (SARS-CoV-2) will put enormous strain on global health, such as bacterial co-infection with COVID-19. Recently (before COVID-19), it has been reported that there has been a decrease in the rate of MRSA infections in some countries [21,25]. Many studies conducted in 2020 found that the patients hospitalized for COVID-19 had received antibiotics even though a secondary bacterial infection was very low [26,27]. The present study did not find an *S. aureus* or MRSA isolate that was resistant to vancomycin. Hence, this antibiotic is still used for severe infections. Similarly, it was reported that MRSA had not been affected by COVID-19, as there was no change in the incidence of MRSA during the pandemic [28]. One similar study found no change in the proportion of MRSA during the COVID-19 pandemic and high resistance to clindamycin [29]. Very few studies suggest that MRSA may emerge as a co-pathogen in COVID-19 infections [30]. The prevalence of MRSA may be associated with prolonged hospital stays, suggesting that it is more likely to be a nosocomial or ventilator-associated infection [30]. No significant difference was found between the incidence and antimicrobial resistance rates of MRSA through the two years (2019 and 2020) (p-value > 0.05). Implement effective infection control measures and changes in hygiene practices that could potentially impact the prevalence and characteristics of MRSA infections. Hence, the present study aimed to determine the prevalence of MRSA strains and their antimicrobial susceptibilities over the years 2019 and 2020 in a tertiary hospital in Makkah city, Saudi Arabia. Overall, emphasizing the strengths of studying MRSA changes before and after 2019 underscores the importance of ongoing research in combating this persistent threat to public health.

There are several limitations to consider in this study. One of these limitations is the data availability and quality; in addition, sampling bias occurred during the selection of specimens. Furthermore, there are challenges in interpreting changes in MRSA rates. Finally, establishing a causal relationship between changes in MRSA and events such as the COVID-19 pandemic after 2019 requires rigorous study design, including statistical analyses, which is a challenge to implement.

## Conclusions

The results of our study showed that the prevalence rates of MRSA did not increase in 2020 when compared to 2019. Vancomycin, linezolid, trimethoprim-sulfamethoxazole, and doxycycline remain susceptible to the positive collected MRSA strains. No significant difference was found between the prevalence and antimicrobial resistance rates of MRSA through 2019 and 2020. Continued research efforts are needed to address this persistent public health threat. Strategies to control the spread of MRSA should include early detection of MRSA and surveillance, even during pandemics.
